# More Than Oestrogen: A Systematic Review of Holistic Strategies for Mental Health Symptoms in Perimenopausal Women

**DOI:** 10.1192/bjo.2026.11232

**Published:** 2026-06-30

**Authors:** Betsy Marina Babu, Kasthuri Padiyan, Asha Dhandapani, Linda Thomas, Sneh Babhulkar

**Affiliations:** 1 ELNFT, London, United Kingdom; 2 MVJ Medical College Research Hospital, Bengaluru, India; 3 BCUHB, Wrexham, United Kingdom; 4 Sheffied Health Partnership University NHS Trust, Sheffield, United Kingdom; 5 NHS Greater Glasgow and Clyde, Glasgow, United Kingdom

## Abstract

**Aims:**

Menopause is when a woman has not had a period for at least 1 year. The median age is 51.Perimenopause is a transitional period associated with fluctuating hormones and heightened vulnerability to mental health symptoms, including depression, anxiety, stress, and sleep disturbance. Fluctuating hormones are noted from the age of 35 years or so. Holistic, non-pharmacological interventions have emerged as potential strategies to address these symptoms, but evidence has not been systematically synthesised.

Aim and Objectives:

1. To review systematically and analyse the evidence on holistic interventions for the management of mental health difficulties in perimenopausal women.

2. To identify various holistic interventions, including psychological, lifestyle, mind-body connection and complementary therapies for perimenopausal mental health symptoms.

3. To evaluate the effectiveness of these interventions for common perimenopausal symptoms.

4. To assess the quality of evidence and to identify the gaps for future research and practice.

**Methods:**

A systematic review of randomised controlled trials was conducted, focusing on holistic interventions for mental health symptoms in perimenopausal women. Databases were searched using MeSH and free-text terms related to perimenopause, mental health, and holistic or complementary therapies. Study quality was assessed using the Cochrane ROB 2 tool.

**Results:**

Twenty RCTs involving approximately 2,700 participants from the USA, Europe, and Asia were included. Interventions were categorised as psychological (CBT, MBCT, MBSR), physical activity-based (yoga,Pilates, tai chi,aerobic exercise), lifestyle modification, or complementary therapies (acupuncture,reiki, music therapy).

CBT-based interventions and mind–body practices consistently improved depression, anxiety, stress, sleep quality, and quality of life. Physical activity-based interventions demonstrated moderate benefits. Evidence for complementary therapies was limited and mixed. Most trials were at low or moderate risk of bias.

**Conclusion:**

Holistic interventions are effective and person-centred approaches for managing mental health symptoms during perimenopause. Psychological and mind-body interventions yielded the most robust benefits. Limitations included heterogeneity of interventions, reliance on self-reported outcomes, and short-term follow-up.

Holistic interventions, particularly CBT, mindfulness, and structured physical activity, improve mental health in perimenopausal women. Future research should focus on larger, culturally diverse trials with standardised outcome measures and longer follow-up to strengthen evidence for clinical guidelines.

